# Statistical analysis of the measured strength parameters of the fresh main intracranial arteries

**DOI:** 10.3389/fbioe.2025.1554674

**Published:** 2025-09-18

**Authors:** Krzysztof Truchel, Krzysztof Wojtas, Radosław Rzepliński, Mikołaj Sługocki, Iwona Łopianiak, Beata Butruk-Raszeja, Krystian Jędrzejczak, Wojciech Orciuch, Paweł Gierycz, Paweł Krajewski, Bogdan Ciszek, Łukasz Makowski

**Affiliations:** ^1^ Faculty of Chemical and Process Engineering, Warsaw University of Technology, Warsaw, Poland; ^2^ Department of Descriptive and Clinical Anatomy, Medical University of Warsaw, Warsaw, Poland; ^3^ Leeds Institute of Cardiovascular and Metabolic Medicine, University of Leeds, Leeds, United Kingdom; ^4^ Department of Forensic Medicine, Medical University of Warsaw, Warsaw, Poland; ^5^ Department of Pediatric Neurosurgery Chair of Neurosurgery, Medical University of Warsaw, Warsaw, Poland

**Keywords:** strength parameters, mechanical properties, maximum Young’s modulus, tensile test, rupture, intracranial arteries, neurovascular diseases, cerebral circulation

## Abstract

This study presents the strength parameters of the major intracranial arteries: basilar artery (BA), anterior cerebral arteries (ACA), both left (LACA) and right (RACA), and middle cerebral arteries (MCA), both left (LMCA) and right (RMCA) and the anterior communicating artery (ACoA) obtained by performing single rupture tensile tests on fresh human biological specimens. The measured parameters included maximum Young’s modulus, E , ultimate strength, $R_{m}$ , rupture strength, $R_{u}$ , and rupture strain, A , allowing the determination of mean values for each artery type. They were also the basis for a multiple comparison analysis showing that ACA and ACoA significantly differ from BA and MCA. The study also showed that the measured strength parameters are directly dependent on the thickness of the arterial wall, and this effect is about 4–5 times greater for the ACA group and 1.5–3 times greater for ACoA than in the BA + MCA group. Finally, a limit value of maximum relative longitudinal strain of 7% was proposed at low risk of rupture during neurovascular procedures. Obtained parameters and findings have potential applications in optimizing neurointerventional devices, tissue engineering of arterial phantoms or tissue scaffolds, and computational simulations in cerebral hemodynamics.

## 1 Introduction

Cerebrovascular diseases represent the primary cause of mortality from cardiovascular diseases, accounting for approximately 30% of all cardiovascular deaths in 2019. This represents a significant increase from the approximately 20% mortality rate observed in 1990 (Tsao et al., 2022; Tejada-Meza et al., 2025).

Existing therapeutic methods do not always provide satisfactory results, and still, common diseases (including hemorrhagic and ischemic stroke) are associated with an unfavorable prognosis. Improved diagnostic methodologies, including angiography, computed tomography, and magnetic resonance imaging, can visualize vascular pathologies at the asymptomatic stage. Therefore, neurology is dynamically developing and looking for solutions to the problems posed by clinicians.

Endovascular procedures within the cerebral and coronary circulation, including thrombectomy in ischemic stroke (Goyal et al., 2016), embolization of arteriovenous malformations (Fleetwood and Steinberg, 2002), stenting of atherosclerotic stenosis (Holmes et al., 2010), and embolization of intracranial aneurysms with coils and stents (Phan et al., 2016), are becoming increasingly commonplace. Despite the efficacy of these procedures, they are not without risk of complications, including restenosis (Bauters and Isner, 1997), stent migration (Diller et al., 2003), and arterial rupture or dissection (Shah et al., 2016). Carrying out interventions requires introducing a set of appropriate catheters, guidewires, balloons, etc., into the intracranial vascular system, which inevitably leads to local mechanical stresses. Critical rupture pressure in major human cerebral arteries has been studied (Ciszek et al., 2013), but the literature lacks data on the tensile strength.

During those interventions, there is a risk of mechanical properties deterioration or even arterial rupture, which can cause significant clinical consequences. Arteries do not obey Hooke’s law due to their highly nonlinear, anisotropic, and viscoelastic mechanical properties observed even for physiological pressures (Maher et al., 2012). The energy resulting from the applied force on a perfectly elastic body is converted into stress, which brings the body back to its original shape when the force is subtracted. For bodies such as arteries, the above mechanical properties cause some of the stored energy in the body to dissipate over time, which means that the body will not return to its original shapes after the force is subtracted (Fung, 1981; Maher et al., 2012). Therefore, arteries lack a single reference state to which they revert following stress subtraction. Instead, the equilibrium state is contingent upon many factors, including the strain history (Fung, 1981). In the context of stent insertion, partial stress relaxation over time will be observed, which is beneficial (Mullins, 1948; Fereidoonnezhad et al., 2016).

Arteries may experience supraphysiological loading during injury or the surgical procedures described above. Therefore, a softening effect is observed, which deteriorates the mechanical properties of the tissue. This effect largely depends on the previous maximum deformation (Fung, 1981; Holzapfel et al., 2002; Maher et al., 2012). When an artery is repeatedly stretched and relaxed, its response to load becomes more predictable, and maximum stresses can be reduced compared to material not subjected to such cycles. This phenomenon is called preconditioning (Fung, 1981; Horný et al., 2010).

The application of stenting following balloon angioplasty is undertaken to prevent the artery wall’s collapse and/or restenosis. However, it should be noted that the risk of arterial rupture is a much more significant consequence of intra-arterial procedures than the changes in mechanical properties themselves. Although circumferential stresses are generated mainly during these procedures, studies show that the ultimate strength of the artery in the circumferential direction is higher than in the axial one (up to two times) (Monson et al., 2008). Moreover, axial stresses can be generated during intravascular procedures such as inserting or withdrawing a catheter or guidewire and during stent delivery, when devices interact with the vessel wall and transmit longitudinal forces. Injury in this mechanism may lead to the pericardial tamponade in case of coronary artery intervention or brain hemorrhage in case of cerebral arteries (Deshpande et al., 2024; Li et al., 2025). Axial tension may also lead to dissection of catheterized vessel (Cloft et al., 2000; Abdelsalam et al., 2024).

In addition to mechanical stress caused by medical procedures such as endovascular interventions, intracranial arteries are also exposed to supraphysiological stress during traumatic brain injury (TBI), particularly in cases involving rapid deceleration, rotational forces, or direct impact (Smith et al., 2003). TBI causes approximately 1.5 million cases annually in the United States, resulting in 50,000 deaths, 230,000 hospitalizations, and long-term disability in 80,000–90,000 individuals (Thurman et al., 1999). These mechanical injuries can lead to diffuse vascular damage, stretching and even rupture of the brain arteries, contributing to intracranial hemorrhage (Ommaya et al., 2002; Smith et al., 2003). During neurosurgical interventions following TBI, vascular reconstruction may involve segmental resection of damaged vessels, followed by direct end-to-end anastomosis or the use of grafts (Wahlgren et al., 2025). Such procedures cause additional axial and circumferential loads, altering the native mechanical environment and requiring an in-depth understanding of the strength parameters of the arteries to prevent postoperative complications such as leakage, pseudoaneurysm, or restenosis.

An essential aspect of arterial strength analysis is the application of the results to engineering research. Currently, many tissue engineering studies are looking for the best materials or printing methods for arterial phantoms or scaffolds (Tetsuka and Shin, 2020; Wang et al., 2020; Tan et al., 2021). These are usually tested, e.g., for biocompatibility with human cells. However, although the results of their stress tests are reported in the literature, due to a lack of data, they are compared only to animal arteries (Illi et al., 2023; Huang et al., 2018; Wang et al., 2020; Lee et al., 2023), or no such comparison is made (Lu et al., 2014; Tang et al., 2015; Łopianiak et al., 2023; 2024; Choudhury et al., 2024). Similarly, such data will be invaluable in selecting the right flexible material for 3D printing using laser measurements (Antonowicz et al., 2023; Jędrzejczak et al., 2023a; 2024a), more accurate reconstruction or validation of arterial mechanics simulations (Holzapfel et al., 2002; 2025), and extending computational fluid dynamics simulations to simulations with dynamic wall deformation (Jędrzejczak et al., 2024b; 2023c; 2023b; Wojtas et al., 2021; Kozłowski et al., 2021; 2022).

This study aimed to examine the mechanical strength parameters of major intracranial arteries: the basilar artery (BA), left and right anterior cerebral artery (LACA, RACA), left and right middle cerebral artery (LMCA, RMCA), and the anterior communicating artery (ACoA). As rupture is most likely to occur during the first ballooning attempt, the arteries were subjected to a single stress increase from an unstressed state to material rupture.

## 2 Materials and methods

The study involved obtaining samples of intracranial arteries and then subjecting them to a quasi-static tensile test. The study protocol was approved by the Ethics Committee of the Medical University of Warsaw (number 194/2024). In the present study, 48 arteries were obtained from 21 cadavers, and 4 of the donors were women. The age of donors ranged from 18 to 81 years (median 54, IQR , 45—65).

### 2.1 Biological material sampling

The study was based on a collection of anatomical specimens from the Department of Descriptive and Clinical Anatomy, Medical University of Warsaw, Poland. The unfixed specimens of the brain were obtained within 5 days of death from the cadavers stored at a temperature of 4 °C–8 °C and prepared with the use of an OPMI Pico microsurgical microscope (Carl Zeiss, Germany), as described previously (Rzepliński et al., 2021). The arteries of the circle of Willis (including BA, MCA, ACA and ACoA) were excised and stored at 4 °C–8 °C in 0.9% saline solution for not longer than 24 h before the experiment. In every case, central nervous system-related causes of death and significant intracranial pathologies were excluded (head trauma, neurodegeneration, cerebral infarction, etc.). Clinical characteristics of the donors were collected.

### 2.2 Quasi-static tensile test

In the quasi-static tensile test conducted on an artery, the specimen is assumed to be a non-porous tube with a constant wall thickness. The artery is fixed between a pair of jaws, subjecting the specimen to tension at a steady speed (5 mm/min). The speed used is consistent with those proposed elsewhere (Fung, 1981), allows the process to be performed in a similar time to stenting (there are no specific procedures for these intravascular procedures; the authors rely on their clinical knowledge), and allows the predominantly elastic behavior of the artery to be mapped. The force used for this, F , concerning initial cross-sectional area, S , expresses the current stress (the first Piola-Kirchoff stress), P :

$$P\left(t\right)=\frac{F\left(t\right)}{S}$$

During the provided tensile test, the engineering strain–relative elongation (in the sense of Eulerian), ε , is calculated based on initial length, l , (for the analyzed arteries, equal to 15 mm) and temporary one, $l\left(t\right)$ :

$$\varepsilon \left(t\right)=\frac{l\left(t\right)-l}{l}$$

By determining the elongation, ε , it is possible to obtain the Young’s modulus, E , which is a parameter that characterizes the material’s elasticity. Given that the most significant risk of arterial rupture occurs at the initial ballooning attempt, the first non-physical stress, the determination of Young’s modulus based on a single instance of stretching is justified. In the case of viscoelastic materials, the course of stress can be described by strain by the following equation (Fung, 1981):

$$\frac{dP}{d\varepsilon }=\beta \varepsilon +E_{0},$$

where $E_{0}$ is constant, and the parameter β represents the rate of increase of Young’s modulus, E , with respect to the increasing tension, P .

However, Monson proposed a more precise solution, where he divides the tensile test range into 3 areas: exponential, liner and quadratic (Monson, 2001). The first is given by equation:

$$P=\frac{B}{A}\left(e^{A\varepsilon }-1\right),$$

where B and A are constants defining the slop of the curve. For the linear part, could be observed:

$$\frac{dP}{d\varepsilon }=\text{const}$$

Combining this approach with the equation given by Fung, it can be deduced that based on the linear area, it is possible to determine the maximum Young’s modulus, E , which in this part of the test has a constant value, which is also the maximum value observed during the entire tensile test. Thus, we obtain:

$${\left.\frac{dP}{d\varepsilon }\right|}_{\text{linear}}\approx E_{0}=E$$

Although the concept of Young’s modulus itself is developed for linearly elastic materials, for pseudoplastic materials it is still useful in describing the slope of the stress-strain curve at a given level of strain and it is used in the literature (Fung, 1981).

**FIGURE 1:**
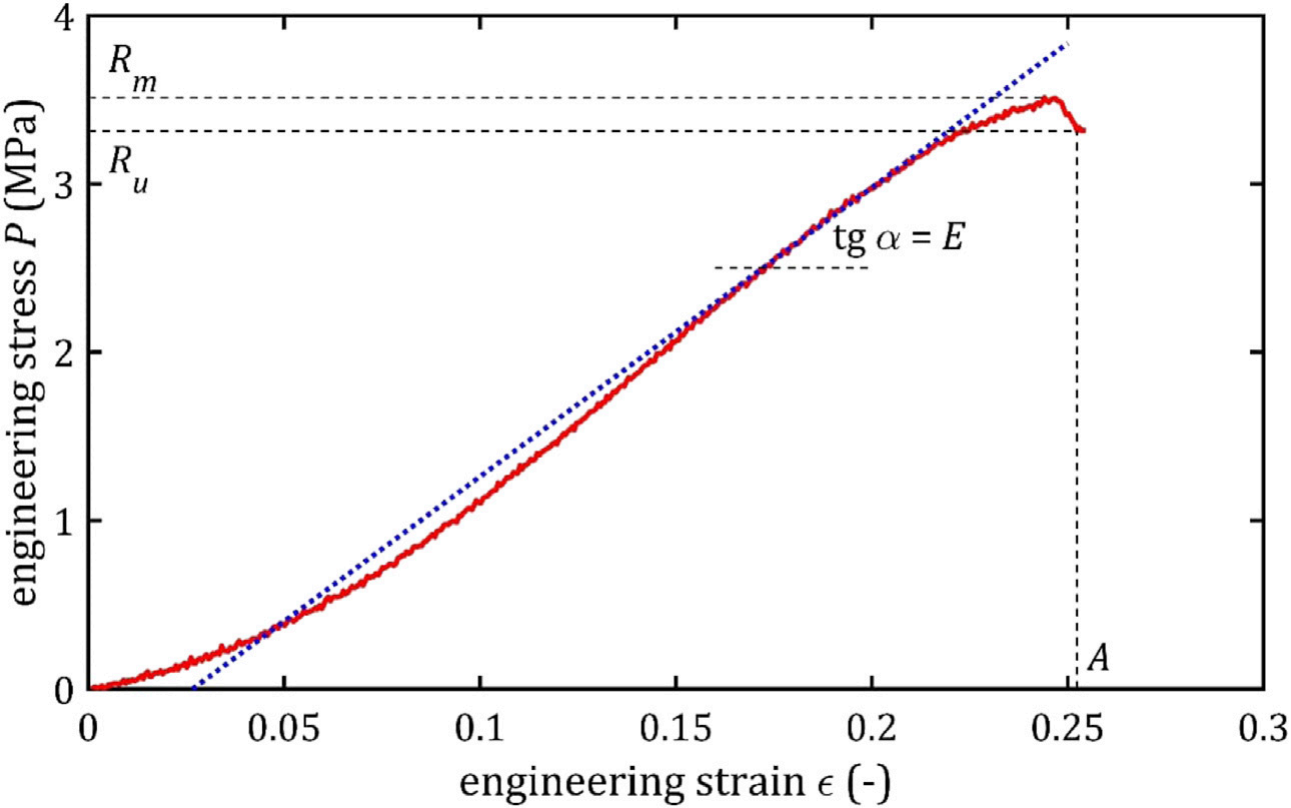
Example of the trend of the quasi-static tensile test of the cerebral artery with marked strength parameters and marked the tangent to the graph and the angle, on the basis of which the maximum Young’s modulus is determined.

The results of the tensile test provide the basis for constructing a graph of engineering stress as a function of engineering strain, $P\left(\varepsilon \right)$ , which, in addition to determining maximum Young’s modulus, allows determination of parameters such as ultimate strength (maximum engineering stress), $R_{M}$ , rupture stress, $R_{U}$ , and rupture strain, A , as shown in Figure 1. In this graph, all three parts of the tensile test described by Monson could be noted.

### 2.3 Strength measurements

**FIGURE 2:**
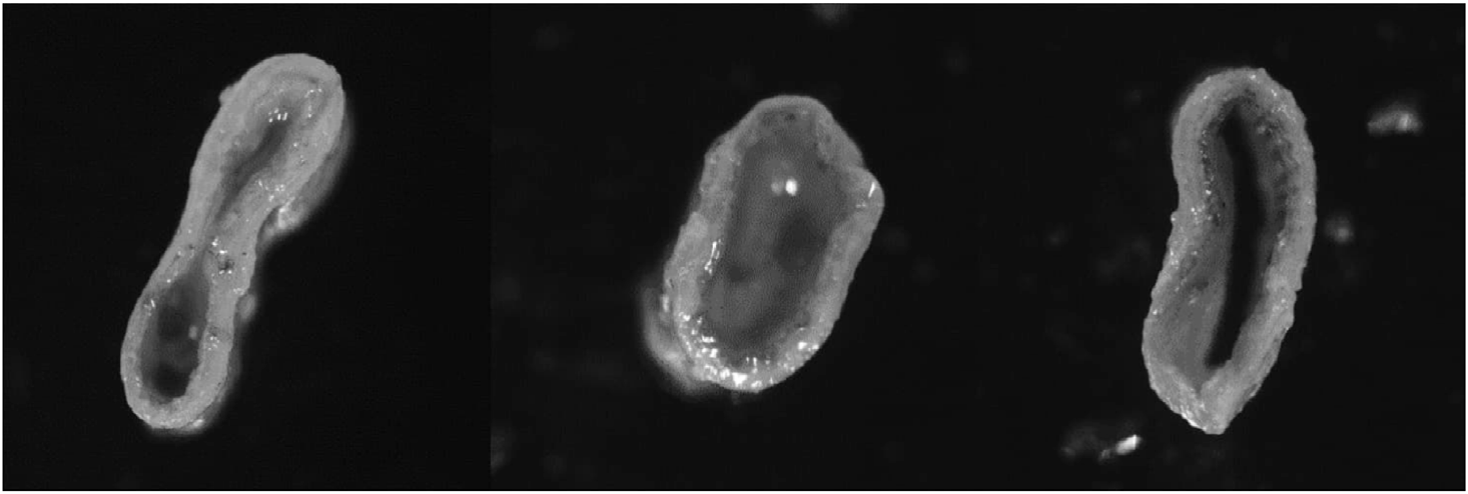
Example images of arterial cross sections.

To determine the cross-sectional area of the artery, a Dantec Dynamics FlowSense 4 M MkII double-frame camera with a resolution of 2048 × 2048 pixels with a Nikon Nikkor AF Micro 60 mm f/2.8D camera lens (supplemented with several spacer rings) was used. A representative slice was taken from each specimen (Figure 2), from which the wall thickness (calculated as the average of a minimum of two circumferential locations) and outer circumference were determined.

The cut and measured specimens with a length of approximately 25–30 mm were subjected to the stretching test in a longitudinal direction. The measurements were performed at room temperature. Samples were stored in saline solution and removed from the saline just before testing to maintain moisture. The central section of each sample, free of branching and wall defects, was placed between pneumatic jaws (distance between jaws = 15 mm). A single-tension test was conducted for each sample using an Instron 3345 device with 50 kN static load cell and associated software Bluehill (v.2), until the specimen reached its failure point.

For this study, we applied the tubular specimen analysis method available in the Bluehill software. The initial (reference) configuration for each sample was defined in the software before testing by inputting the following measured parameters: wall thickness, outer diameter, gauge length (15 mm, i.e., the distance between the grips).

Samples were positioned in the grips such that the central, uniform portion of the vessel—free of visible defects or irregularities—was included within the gauge length. Because arteries in the human body are constantly subjected to certain stresses that the dissected sample did not experience, the artery was positioned in the jaws to approximate its natural tension. Due to the lack of data on specific stresses, this positioning was based on the medical experience of team members who had previously dissected these specimens. The samples remained in this position for about 10 s for preconditioning, and then the force and displacement sensors were zeroed to ensure accurate acquisition of mechanical data.

Throughout the entire testing process, the specimens were kept hydrated. They were stored in physiological saline solution until the moment of testing, and due to the short duration of each test (typically 1–2, maximum 3 min until failure), the hydration state was maintained during the entire measurement. During testing, samples were recorded to detect where the break occurs. The results of those tests in which the specimen slid out, broke at the jaw, or the exact location of the break could not be located, were rejected.

**FIGURE 3:**
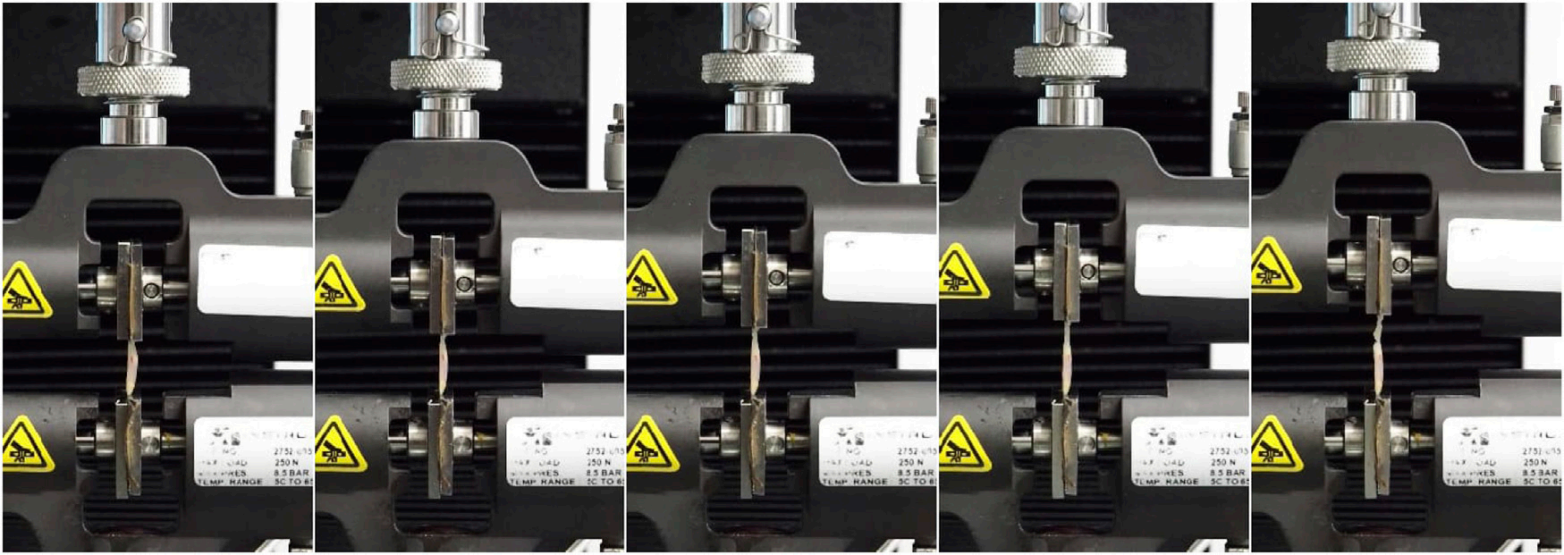
The run of the single tensile test by the Instron 3345 device from the initial moment (first photo) in 20 s increments points to the final rupture moment (last photo).

The tensile process described above is shown in Figure 3.

### 2.4 Statistics analysis

Due to the potential for inconsistencies in sample preparation and tensile testing, some results exhibited a notable difference compared to others. For groups of individual arteries, the coarse errors were identified as the results falling outside the extreme area in the Box-Whisker chart, that is, the area $\left[Q_{1}-1.5\;IQR,Q_{3}+1.5\;IQR\right]$ , where IQR is the interquartile range $\left[Q_{1},Q_{3}\right]$ .

The question arises whether the results obtained for each type of artery are significantly different and should be considered for each artery separately. For this reason, an analysis of variance (ANOVA) was performed to test the null hypothesis (St and Wold, 1989):

$$H_{0}:{\bar{y}}_{\text{BA}}={\bar{y}}_{\text{LACA}}={\bar{y}}_{\text{RACA}}={\bar{y}}_{\text{LMCA}}={\bar{y}}_{\text{RMCA}}$$

where the expected values for each group of arteries - the group averages of each strength parameter, $\bar{y}$ , were checked for equality. The analysis was conducted on a=5 groups of arteries with $m_{i}$ observations each (the number of observations in individual groups was not equal).

The impact of the artery type on the parameter under investigation is amplified when there is more significant variability and thus intergroup variance (mean square for artery effect, $MS_{A}$ ) relative to the error variability or residual variance within groups (mean square for errors, $MS_{E}$ ). Consequently, a test statistic can be defined as follows:

$$F_{L}=\frac{MS_{A}}{MS_{E}},$$

where the variance estimator, $MS_{A}$ , is the sum of the squares of the variation in arterial type, $SS_{A}$ , related to its number of degrees of freedom, ${DF}_{A}$ :

$$MS_{A}=\frac{SS_{A}}{DF_{A}}$$

$$SS_{A}=\sum_{i=1}^{a}m_{i}{\left({\bar{y}}_{\left(i.\right)}-{\bar{y}}_{\left(..\right)}\right)}^{2}$$

$$DF_{A}=a-1$$

In comparison, the residual variance estimator, defined as $MS_{E}$ , is the sum of squares of group errors, SSA, related to its number of degrees of freedom, ${DF}_{E}$ :

$$MS_{E}=\frac{SS_{E}}{DF_{E}}$$

$$SS_{E}=\sum_{i=1}^{a}\sum_{j=1}^{m_{i}}{\left(y_{\left(ij\right)}-{\bar{y}}_{\left(i.\right)}\right)}^{2}$$

$$SS_{E}=\sum_{i=1}^{a}\sum_{j=1}^{m_{i}}{\left(y_{\left(ij\right)}-{\bar{y}}_{\left(i.\right)}\right)}^{2}$$

The probability level corresponding to the Fisher test statistic, p , indicates the potential for errors in rejecting the null hypothesis. When p -value exceeds the pre-established significance level of the test, α , then the $H_{0}$ hypothesis is rejected.

In this case, it is also possible to identify which groups of arteries exhibit statistically significant differences. Thus, it could be conducted to evaluate the significance of the contrasts between each pair, following a null hypothesis analysis:

$$H_{0}:{\bar{y}}_{k}={\bar{y}}_{l},$$

where k and l are the following pairs of arterial groups.

There are several test statistics to compare different groups of results with varying degrees of power and to test the significance of any contrasts. One of these is Fisher’s Least Significant Difference (LSD) test (Williams and Abdi., 2010), which has one of the highest levels of power, making it very likely to identify the required differences correctly (Seaman et al., 1991). The formula gives the test statistic:

$$T=\frac{{\bar{y}}_{\left(k.\right)}-{\bar{y}}_{\left(l.\right)}}{SE_{L_{i}}}$$

The contrast standard error takes the value:

$$SE_{L_{i}}=\sqrt{MS_{E}\sum_{j=1}^{a}\left(\frac{1}{m_{j}}\right)}$$

This statistic has the t-Student distribution with ${DF}_{E}$ degrees of freedom. The higher the value of the above statistic, the lower the corresponding probability level.

The condition for rejecting the above hypothesis is that the inequality is satisfied:

$$\left|{\bar{y}}_{\left(k.\right)}-{\bar{y}}_{\left(l.\right)}\right|\geq t_{k,\alpha ,DF_{E}}SE_{L_{i}},$$

where $t_{k,\alpha ,DF_{E}}$ is the critical value of the random variable of the t-Student distribution for the applied significance level, α .

A multivariate regression analysis can be conducted to investigate the potential impact of factors such as age, sex, and geometric parameters of arteries on the obtained strength parameters.

A function is introduced in multiple regression and generalized linear models, where n repetitions and p variables are considered.

A generalized linear model is employed to analyze data from n repetitions and p variables, which is represented by a function:

y=xb+e,

where x is input value matrix:

$$x=\left[\begin{array}{cccc} 1 & x_{\left(1\right)1} & \ldots & x_{\left(1\right)p}\\ 1 & x_{\left(2\right)1} & \ldots & x_{\left(2\right)p}\\ \vdots & \vdots & \ddots & \vdots \\ 1 & x_{\left(n\right)1} & \ldots & x_{\left(n\right)p} \end{array}\right],$$

y is the matrix of the output values obtained in the experiment:

$$y^{\prime }=\left[y_{\left(1\right)},y_{\left(2\right)},\ldots y_{\left(n\right)}\right],$$

b is the vector of the regression coefficients:

$$b^{\prime }=\left[b_{0},b_{1},\ldots b_{p}\right]$$

and e is the vector of random observation errors:

$$e^{\prime }=\left[e_{\left(1\right)},e_{\left(2\right)},\ldots e_{\left(n\right)}\right].$$

In searching for an optimal regression model, it is necessary to determine which of the identified regression coefficients is significant, significantly different from zero. This can be achieved by comparing the value of the coefficient with the imprecision of its determination, which is measured by the standard deviation:

$$t=\frac{b}{s_{b}},$$

where

$$b={\left(x^{\prime }x\right)}^{-1}x^{\prime }y$$

$$s_{b}={\left(x^{\prime }x\right)}^{-1}\frac{y^{\prime }y-b^{\prime }x^{\prime }y}{n-p-1}.$$

The statistic is derived from the t-Student’s distribution. As the value of the test statistic increases and the corresponding probability level decreases, the regression coefficient under analysis becomes more significant. When the p -value for the specific parameter exceeds the preestablished significance level of the test, α , then the impact of this factor is negligible and the analysis should be carried out again, excluding this parameter.

All the statistical calculations described above were performed using the Statgraphics 19 software from Statgraphics Technologies, Inc.

## 3 Results and discussion

All stress-strain curves for all studied arteries are presented in Figures 4–9. Figures 10–13 illustrate the strength parameters for all analyzed cerebral artery types, and Table 1 presents their mean values with standard deviation. The detailed characteristics of all arteries are available in the Supplementary Table S1.

**FIGURE 4:**
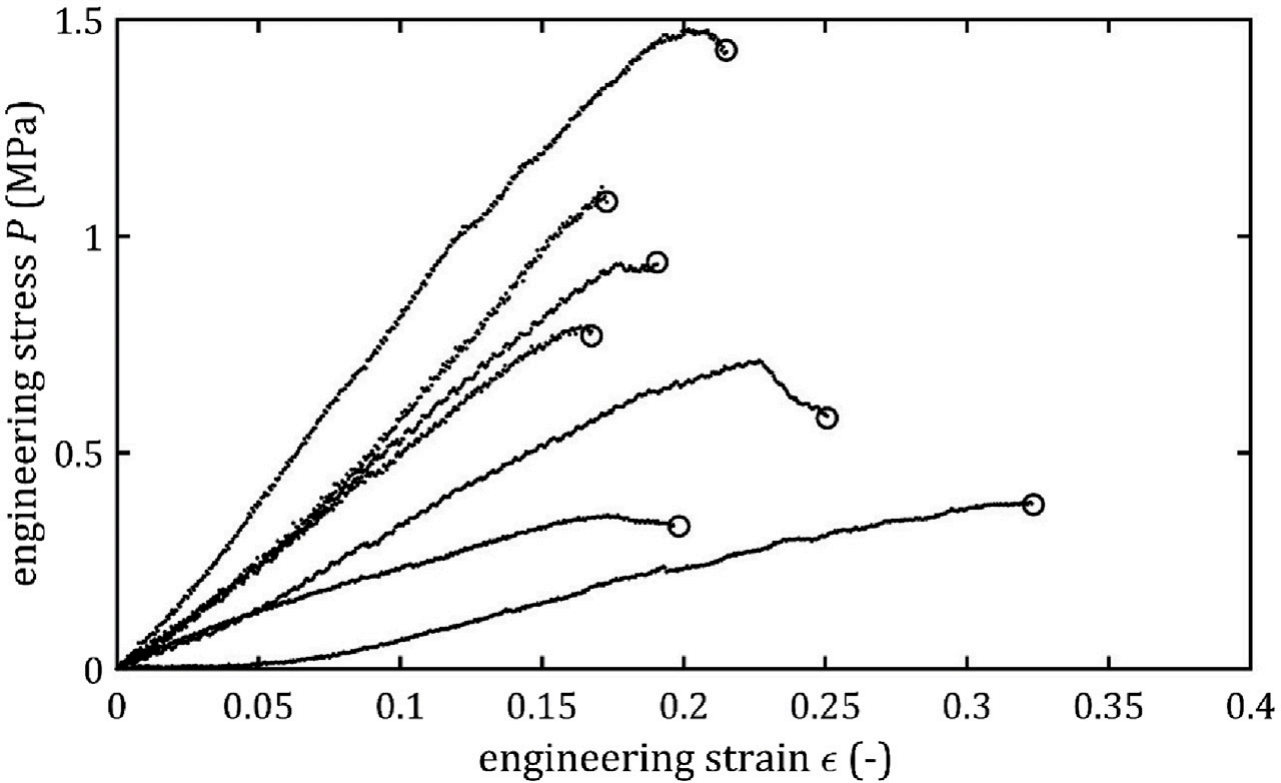
Stress-strain curves from quasi-static tensile tests for BA.

**FIGURE 5:**
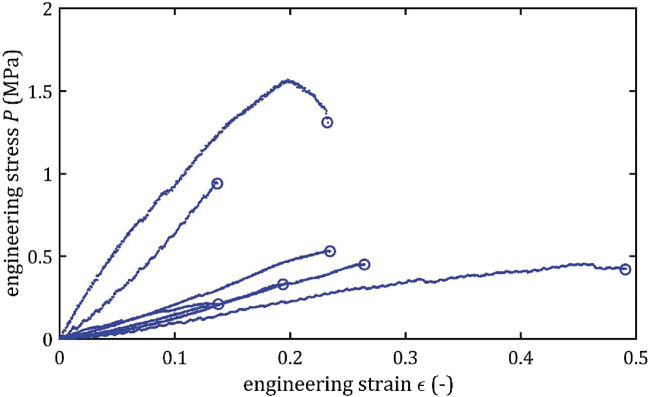
Stress-strain curves from quasi-static tensile tests for LMCA.

**FIGURE 6:**
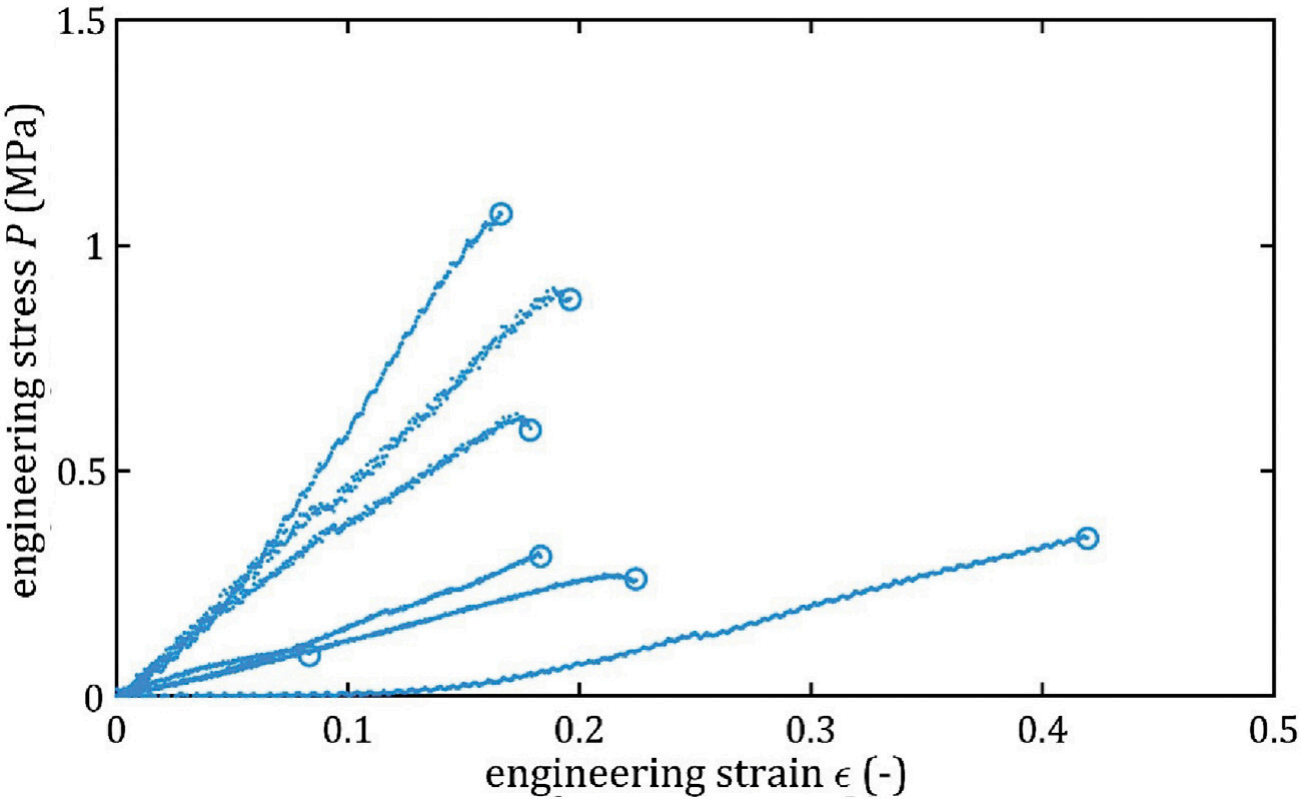
Stress-strain curves from quasi-static tensile tests for RMCA.

**FIGURE 7:**
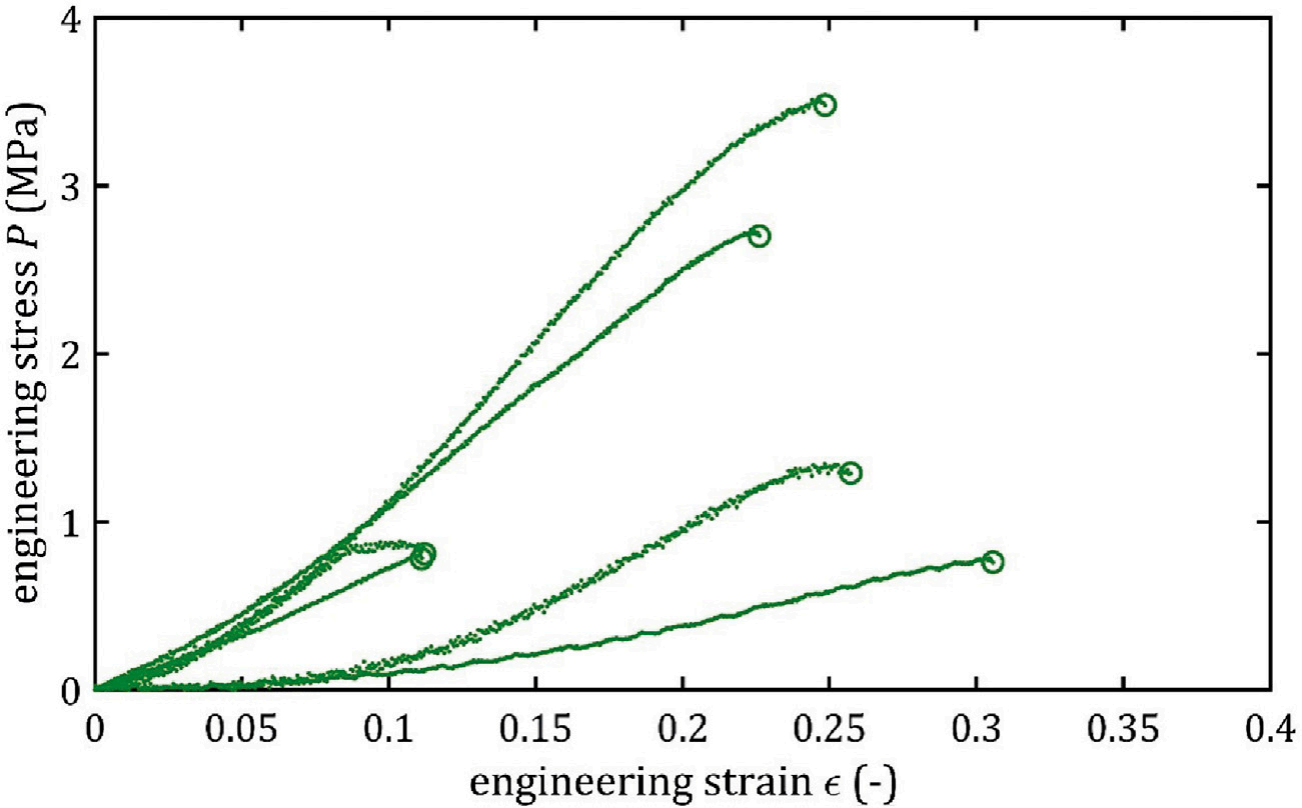
Stress-strain curves from quasi-static tensile tests for LACA.

**FIGURE 8:**
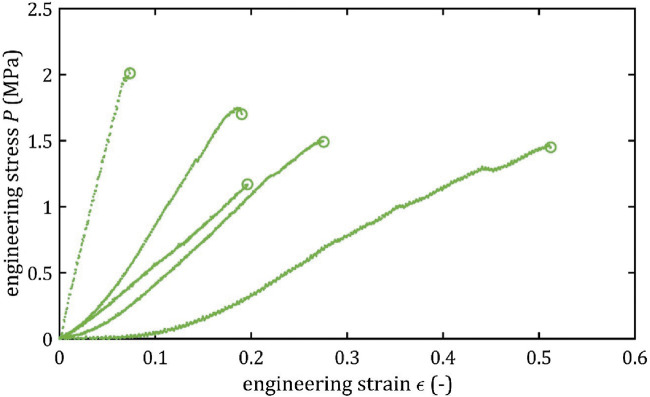
Stress-strain curves from quasi-static tensile tests for RACA.

**FIGURE 9:**
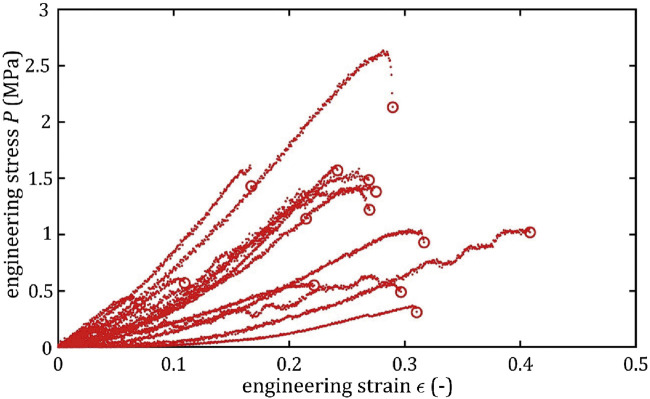
Stress-strain curves from quasi-static tensile tests for ACoA.

**FIGURE 10:**
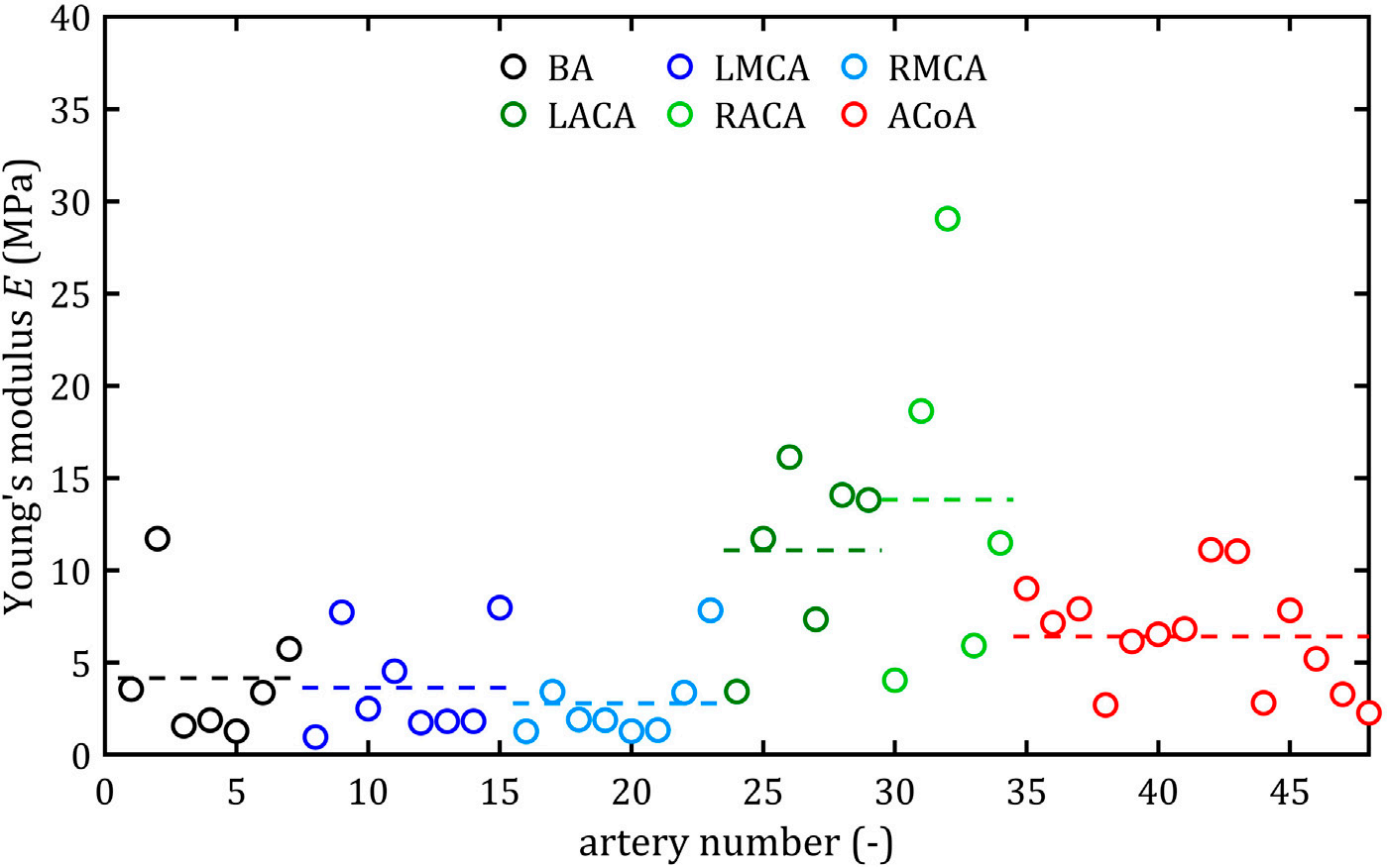
Maximum Young’s modulus, *E*, values for different cerebral arteries (dots represent the results of each sample, the dashed lines present the average value of a specific type of artery).

**FIGURE 11:**
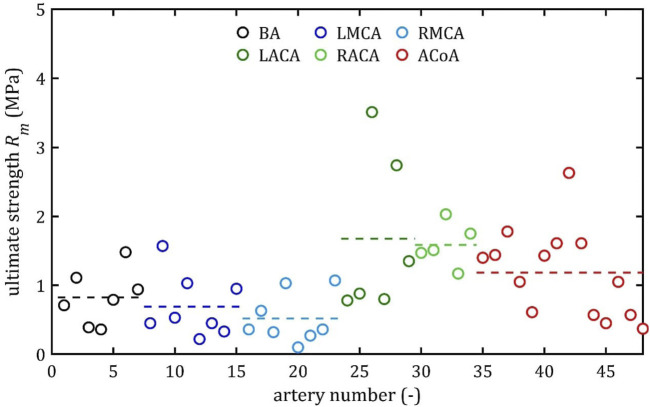
Ultimate strength, *R*_*m*_, values for different cerebral arteries (dots represent the results of each sample, the dashed lines present the average value of a specific type of artery).

**FIGURE 12:**
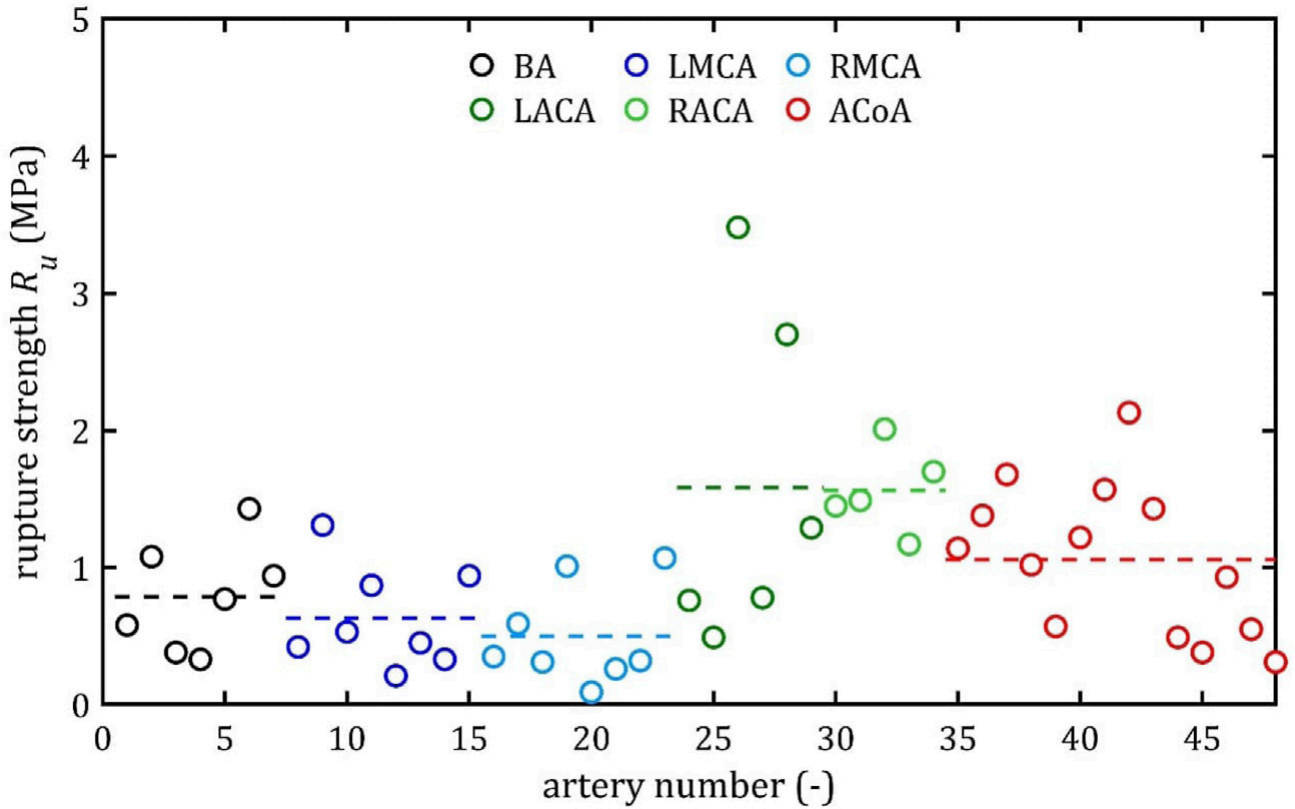
Rupture stress, *R*_*u*_, values for different cerebral arteries (dots represent the results of each sample, the dashed lines present the average value of a specific type of artery).

**FIGURE 13:**
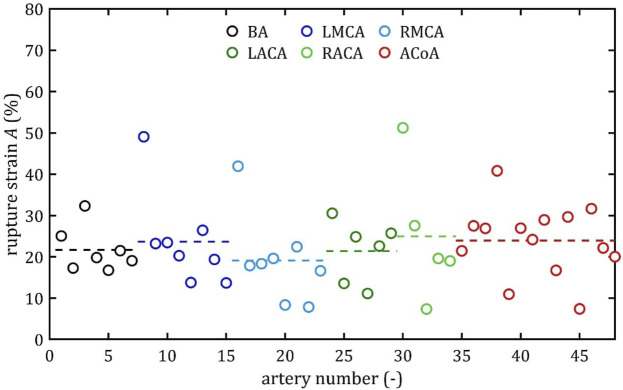
Rupture strain, *A*, values for different cerebral arteries (dots represent the results of each sample, the dashed lines present the average value of a specific type of artery).

**TABLE 1:** Mean values and standard deviation, *P*, of tensile test results for different types of cerebral arteries.

Artery	*N* (−)	$\bar{E}$ (MPa)	*σ* (*E*) (MPa)	${\bar{R}}_{m}$ (MPa)	*σ* (*R*_*m*_) (MPa)	${\bar{R}}_{u}$ (MPa)	*σ* (*R*_*u*_) (MPa)	$\bar{A}$ (%)	*σ* (*A*) (%)
BA	7	4.17	3.66	0.83	0.40	0.79	0.40	21.69	4.35
LMCA	8	3.64	2.80	0.69	0.45	0.63	0.37	23.66	11.22
RMCA	8	2.80	2.22	0.52	0.36	0.50	0.36	19.13	10.59
LACA	6	11.09	4.79	1.68	1.17	1.58	1.22	21.40	7.52
RACA	5	13.83	10.23	1.59	0.32	1.57	0.31	24.96	16.36
ACoA	14	6.42	2.91	1.18	0.64	1.06	0.55	23.96	8.56

E – maximum Young’s modulus; $R_{m}$ – ultimate strength; $R_{u}$ – rupture stress; A – rupture strain.

### 3.1 Strength parameters

Mean value of maximum Young’s modulus, $\bar{E}$ , was the lowest in the MCA group (2.80 MPa for the RMCA and 3.64 MPa for the LMCA) and the highest in the ACA group (13.83 MPa for the RACA and 11.09 MPa for the LACA). Similarly, mean ultimate strength, ${\bar{R}}_{m}$ , and mean rupture stress, ${\bar{R}}_{u}$ , were the highest in the RACA group and amounted to 1.59 MPa and 1.57 MPa, respectively. Mean rupture strain, $\bar{A}$ , ranged from 19.13% for the RMCA to 24.96% for the RACA.

As the results show, each artery leads to different average values. Monson provided measured values of cerebral arteries, without dividing them into specific arteries. For quasi-static tensile test for small arteries on the surface of the temporal lobe, mechanical parameters are E=21.42 MPa , $R_{u}=4.14\text{ MPa}$ (Monson et al., 2003), but in different paper for MCA there are: E=18.18 MPa , $R_{u}=3.23\text{ MPa}$ (Monson et al., 2005). Those values are quite different from those obtained in tests conducted in this paper, a possible reason for which is the stretching time. The tests conducted in the present study were longer (about 0.5–2 min) than those of Monson (a few seconds), making the material behave more elastic due to its viscoelastic properties and leading to lower values of maximum Young’s modulus values and higher stresses (Fung, 1981; Monson et al., 2003). Such differences have been observed, for example, in coronary arteries tested at different elongation rates (Karimi et al., 2017) and cerebral bridging veins (Sánchez-Molina et al., 2021), where differences in ultimate strength between the low/medium elongation rate and high one were even more than 3 times lower. Bearing in mind this observation of fact and remembering that these values are different for different types of arteries, it can be assumed with a high degree of certainty that the results obtained are correct, since for lower strain rates the results obtained were about twice as low.

However, it can be seen that the MCA strength parameters differ from the results of other intracranial artery, suggesting that the differences between the types are significant, with the MCA itself leading to lower values (as in this study). In the literature, there are studies based on human intracranial arteries that lead to much lower values of $R_{u}$ , e.g., 1.06–1.34 MPa, which fit in range of obtained data. Very similar studies conducted for animal arteries led to $E\in \left[4,13\right]\text{ MPa}$ , $R_{u}=\left[0.5,5.0\right]\text{ MPa}$ , $\lambda_{u}=\left[1.4,2.0\right]$ for wall thickness in range $\delta =\left[0.07,0.14\right]\text{ mm}$ and extemal diameter $D=\left[0.6,1.2\right]\text{ mm}$ for sheep’s MCA’s (Nye et al., 2017) and $E\in \left[0.5,6.5\right]\text{ MPa}$ for $D=\left[0.22,0.30\right]\text{ mm}$ for rat (Coulson et al., 2002). Although the geometry dimensions are smaller than in human arteries, the values of the mechanical variables are similar for those in this study.

It is also noteworthy that the relative positions of the average strength, both ultimate, $R_{m}$ , and rupture, $R_{u}$ , correspond to the relative positions of the rupture pressures of specific arteries (Lombarski et al., 2022). This further corroborates the accuracy of the obtained results. On this basis, it is possible to acknowledge the validity of the research carried out, while additionally noting the need for a more thorough study of the effect of strain rates on the results obtained.

Both the mean and the individual results demonstrate that the difference between ultimate, $R_{m}$ , and rupture, $R_{u}$ , strength is negligible (in paired t-test p -value = 0.0083, the mean of the relative differences between these parameters is less than 10%), and in half of the samples the error was less than 5%. However, even before they rupture or reach the point of maximum stress, the assumption that their mechanical properties remain completely unchanged is incorrect. Although arteries are generally viscoelastic (Fung, 1981) and there is some stress relaxation when stretched below the failure, more recent studies show that even excessive stretching below the failure can induce permanent, irreversible changes in arterial mechanics (Holzapfel et al., 2002). This means that there is a risk of permanent damage even below the stress that will cause permanent deformation in the passive response. Nevertheless, it is no less important to know ultimate stress, due to which it is possible to accurately model the mechanical deformation of arteries and provide a reference point for clinicians.

As can be observed in the Figures 10–13, the results obtained for the different artery types in relation to the maximum Young’s modulus, E , ultimate strength, $R_{m}$ , and rupture strength, $R_{u}$ , are similar. The results for BA, MCA, and ACoA are much more repeatable, as evidenced by standard deviation values approximately twice as small as those for ACA (Table 1). The results for rupture strain, A , exhibit lower repeatability, with observed differences between groups being smaller and standard deviation values higher than for other strength parameters. Notably, minimum results were observed at a similar level of around 7% for each artery type.

### 3.2 Multiple comparisons

**FIGURE 14:**
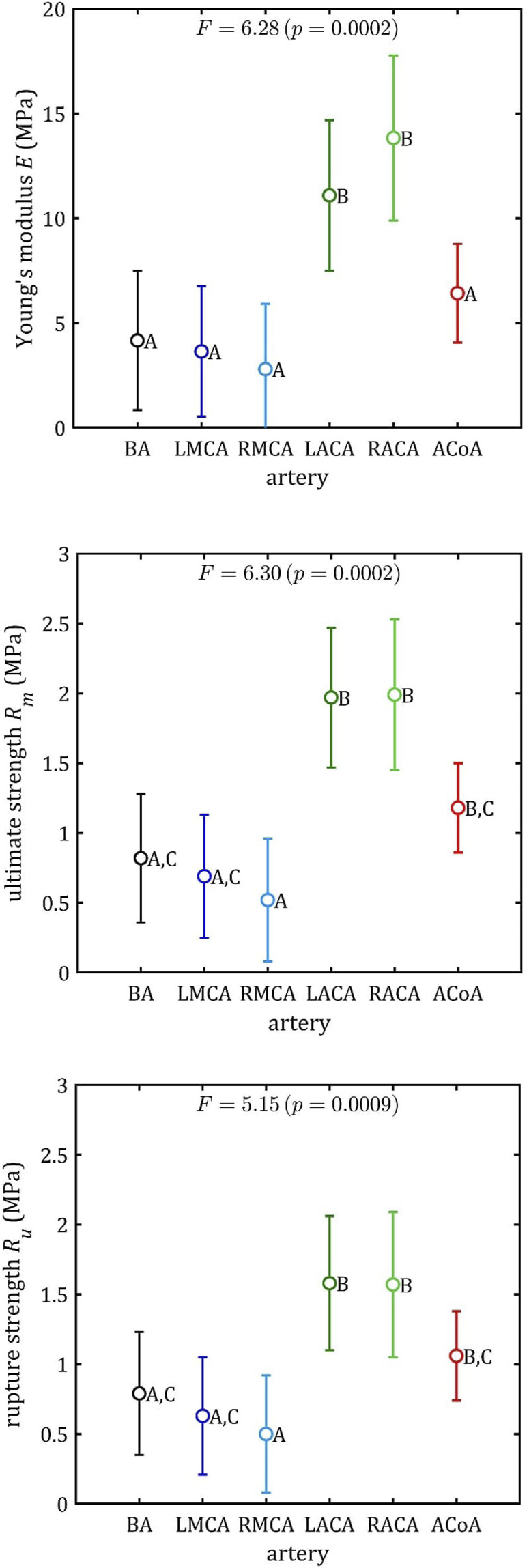
Results of multiple comparisons (means, 95% percent LSD intervals and homogeneous group: A–C) for strength parameters.

The results of the multiple comparisons between the different arterial groups are presented in Figure 14 (specific data available in Supplementary Tables S2–S3). The graphs show the strength parameters’ mean values and the 95% LSD intervals. On this basis, the Roman letters indicate homogeneous groups for which there are no significant differences in result.

The ACA demonstrates notable differences in each of these three parameters (maximum Young’s modulus, E , ultimate strength, $R_{m}$ , and rupture strength, $R_{u}$ ) when compared to other arteries. Furthermore, a comparison of ACoA with BA and MCA reveals differences in stress levels. Accordingly, the following three groups of arteries can be defined for further consideration: BA + MCA, ACA and ACoA.

This conclusion is less evident in the case of rupture strain, A ,. The p -value is 0.15 which is higher than 0.05 (95% confidence level). Based on these values, the probability of significant differences in strain results between groups is low. This means that the moment of rupture itself does not depend on the type of artery, and the limit of 7%, discussed earlier, can be the same for all analyzed intracranial arteries.

### 3.3 Linear regression

**FIGURE 15:**
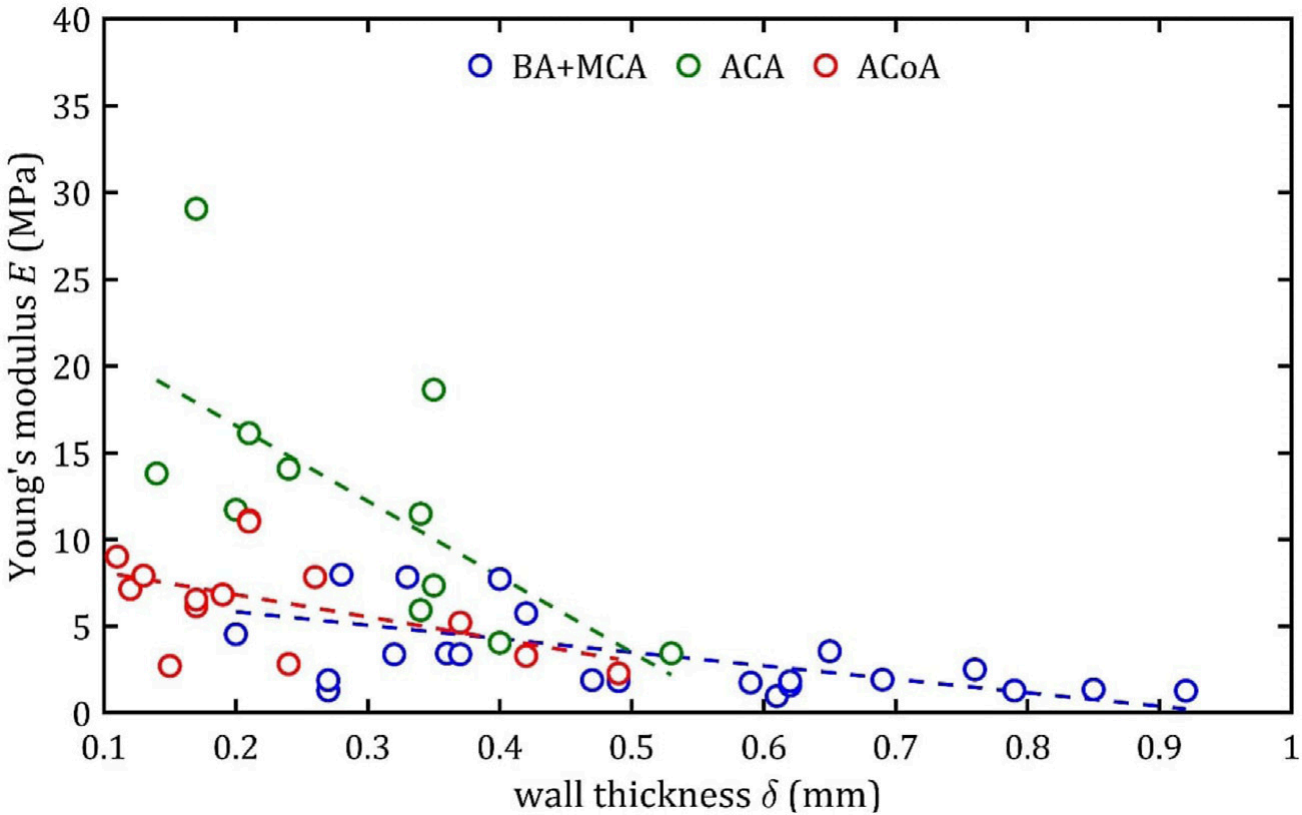
Trends of changes in Young’s module, E , value with change in wall thickness, δ , for BA + MCA, ACA and ACoA groups.

**FIGURE 16:**
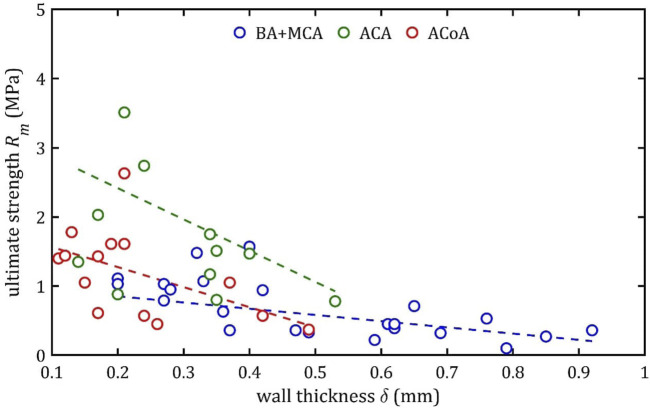
Trends of changes in ultimate strength, $R_{m}$ , value with change in wall thickness, δ , for BA + MCA, ACA and ACoA groups.

**FIGURE 17:**
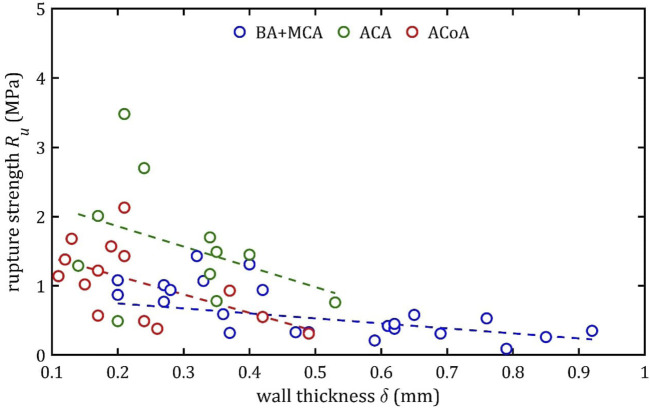
Trends of changes in rupture strength, $R_{u}$ , value with change in wall thickness, δ , for BA + MCA, ACA and ACoA groups.

Based on this division (BA + MCA, ACA, and ACoA groups), multiple linear regression could be performed (Figures 15–17). These statistical calculations include testing the influence of several variables on all the strength parameters analyzed. Analysis revealed that strength parameters were related only to arterial wall thickness, δ , whereas relations with age, sex, and outer diameter were not detected ( p -value much higher than 0,05).

According to classical principles of continuum mechanics, the stress acting on the wall of a vessel subjected to internal pressure is inversely proportional to the thickness of the wall (Fung, 1994). This means that a vessel with a thicker wall will experience less stress per unit area for the same pressure value, which translates into greater resistance to mechanical failure and delamination. Hence, increasing the thickness of the arterial wall is a natural adaptive mechanism that allows the vessels to cope with higher pressures and mechanical loads, and changes in wall thickness are a key component of vascular remodeling that affect both the strength and elasticity of arteries (Humphrey, 2002).

A paper on the mechanics of the aorta also noted a similar dependence of stiffness on the thickness of the wall (Chen et al., 2024). In consideration of the aforementioned works and others in the field (Fung, 1981; Humphrey, 2002), it is evident that the thickness of the wall is contingent upon factors such as age, individual characteristics, medical history, and health status. Consequently, these parameters also indirectly affect mechanical properties, although their influence is difficult to determine unequivocally.

It is worth considering the lack of statistical influence of other analyzed aspects on mechanical properties. The present linear regression tests examined parameters clinicians can easily measure before or during surgical procedures. The analysis showed that gender had no significant effect on the parameters studied. However, trends analogous to those observed for wall thickness, δ , were also evident for age, although to a significantly smaller extent. This is probably related to the fact that arterial wall thickness, δ , is also influenced by age (Cogswell et al., 2019), as well as medical history and lifestyle. The influence of wall thickness, δ , is more pronounced than that of age, suggesting that the thickness influences the observed parameters. It is additionally worth noting that the small effect of age on the parameters studied may be due to the fact that a large part of the samples came from middle-aged cadavers (40–60 years old), and there were few young/older individuals. This article focuses on examining the effect of wall thickness, which turns out to be more important than age, although the study itself does not reject the notion that there is a dependence of mechanical parameters of arteries on age.

It should be noted that the observed relationship between wall thickness, δ , and strength parameters, including Young’s modulus, E , does not imply a direct causal relationship. Young’s modulus, E , is a material property that reflects the microscopic composition of the vessel wall (Lakatta and Levy, 2003; Vatner et al., 2021). However, there are studies suggesting that wall thickness, δ , a geometric parameter, can serve as a surrogate marker of cumulative structural remodeling in the arterial wall (Fung, 1981). Thickness changes are often accompanied by age-related microstructural changes - such as elastin fragmentation, collagen accumulation and crosslinking - that are known to affect the stiffness and risk of arterial damage. Therefore, although wall thickness, δ , is not a major determinant of Young’s modulus, E , it may correlate with it indirectly due to unmeasured histological features and other parameters not directly dependent on patient age.

It is also worth recalling ACoA differs from the ACA, MCA, and BA in several aspects, including embryological origin, high anatomical variability, and increased susceptibility to aneurysm formation (Krystkiewicz et al., 2021; Bonasia et al., 2022). ACoA often presents hypoplasia, aplasia, duplication, and fenestrations (Krzyzewski et al., 2014) and it remains the most frequent site of intracranial aneurysms, accounting for up to 23%–40% of cases (Pahwa et al., 2022). The current study encountered diverse arterial structures, ranging from simple to complex configurations, including those with multiple branches and bifurcations. Moreover, the analysis revealed no significant impact of the structure on the outcomes, and ACoA measurements led to the lowest standard deviation values among all arteries.

An increase in diameter results in a decrease in maximum Young’s modulus, E , ultimate strength, $R_{m}$ , and rupture strength, $R_{u}$ , This observation is consistent across all the groups, yet a notable distinction can be observed in the slope. Assuming the group with the smallest slope, BA + MCA, as the baseline, it can be observed that ACA demonstrates slopes that are up to 4–5 times higher, and ACoA exhibits slopes that are 1.5–3 times higher.

This phenomenon may be due to different arterial wall layers’ proportions, with relatively thicker tunica media in bigger arteries, making them less stiff. In the case of the ACA, the different composition is a consequence of embryological development (Lasjaunias et al., 2006).

**FIGURE 18:**
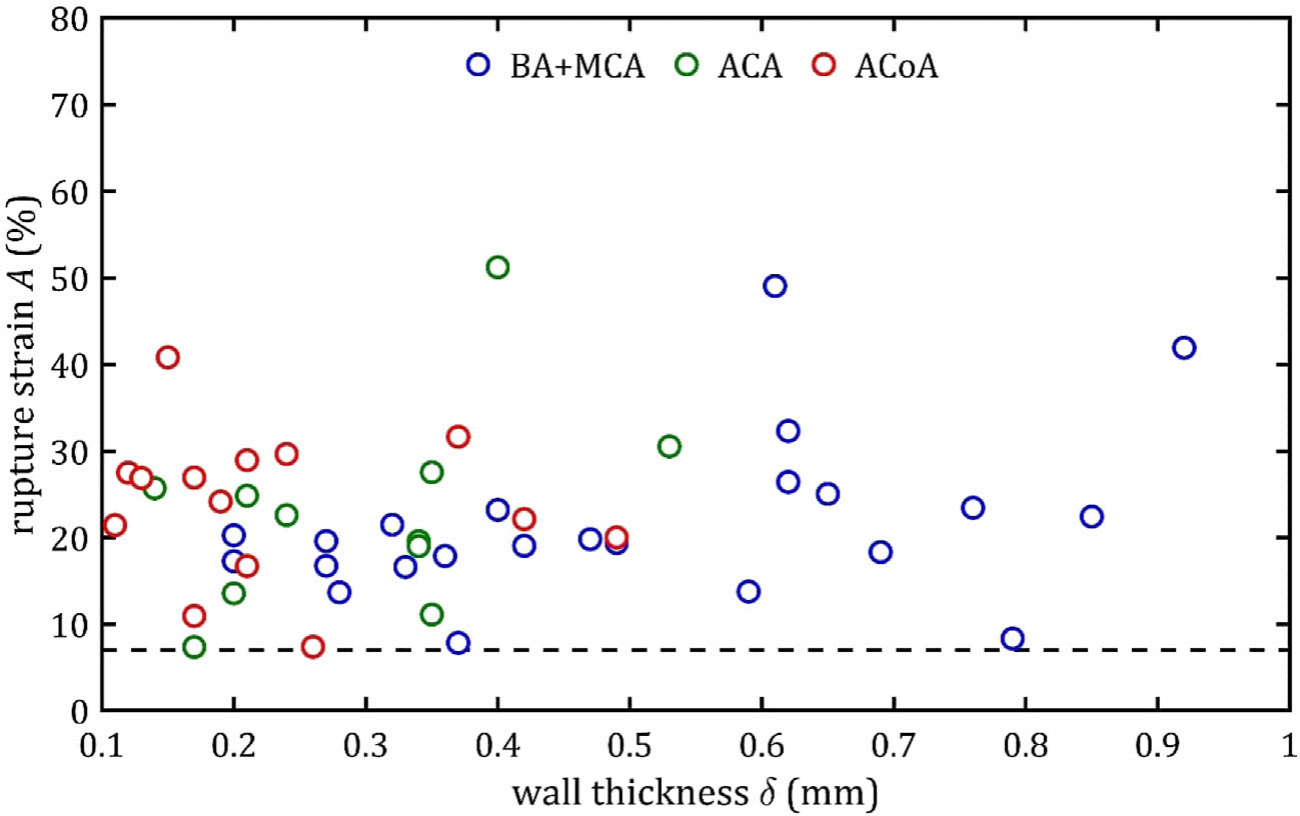
Trends of changes in rupture strain, A , value with change in wall thickness, δ , for BA + MCA for BA + MCA, ACA and ACoA groups (the black dashed line indicates the minimum value for all groups).

It is noteworthy that rupture at 7% elongation was found for different types of arteries and for different wall thicknesses (mainly for small ones below 0.4 mm but even for large thicknesses around 0.8 mm) (Figure 18). This indicates that the presented limit should be independent of wall thickness. Moreover, it should be borne in mind that in a pathological condition (e.g., infiltration by a tumor or inflammation) the strength parameters may be altered and practice shows that arteries are more fragile. The proposed limit may then be inappropriate. Moreover, there are several small branches of BA, ACA and MCA called the perforating arteries that at branch points may alter the mechanical properties of arterial wall (Rzepliński et al., 2022; 2023b; Kwiatkowska et al., 2023). The small arteries are hardly visible in standard clinical imaging techniques (Rzepliński et al., 2023a), therefore performing neurointerventions require special caution. Taking all of the above aspects into account. It is proposed that the 7% limit should be a benchmark for clinicians in the case of intracranial arteries, indicating that longitudinal stretching beyond this limit is associated with an increasing likelihood of irreversible consequences or even rupture. Of course, this is not a safe limit at which there is no risk of deterioration of mechanical properties or permanent damage to the artery. Nevertheless, the value itself can stand as some indication to clinicians that even with such small longitudinal deformations there is a risk of treatment failure.

Applying this limit in practice means that stretching should be avoided during intracranial procedures when arteries are manipulated and thus stretched (e.g., mechanical thrombectomy) or bypassed. For the analyzed arteries, whose length is in the order of a few millimeters, the possible maximum stretching is only a few millimeters (Rhoton, 2000).

Most arterial procedures are performed using endovascular surgery, which employs tensile force in a circumferential direction. The structure and layout of the fibers result in different mechanical parameters in the axial and circumferential directions, with the higher circumferential strength (Fung, 1981). It is important to acknowledge that axial stresses are generated within the artery even in circumferential tension. Consequently, when examining the deformation of endovascular procedures, it is also essential to consider the maximum safe limit of axial elongation.

### 3.4 Limitations

The study has several limitations. The specimens were obtained from 21 brains, but conducting anatomical specimens-based studies is always limited by specimens’ availability. It is important to consider that when basing experimental results on biological material, the exact history of diseases, health conditions or genetic predispositions is not entirely known. This may affect the scatter or value of the parameter results obtained and the conclusions of the statistics performed.

It is also worth noting the time for testing the arteries after the occurrence of death. It was a maximum of 5 days, which was a direct result of medical procedures and the possibility of testing biological materials. However, a sizable portion of the samples were 1–2 days old. The study did not note any significant differences between the results for arteries with different collection times. An important fact is that intracerebral arteries consist mainly of elastin and collagen (the first type), which do not degrade fast enough to affect the results of the tests performed (Fung, 1981; Li et al., 2020). This is also in line with observations that indicate the possibility of mechanical testing of biological materials up to 7 days after in accordance without any change in strength parameters (Tuttle et al., 2014).

The technical limitation of the study was the accuracy of measuring the vessel diameter and thickness based on microscopic images. The cut tissue fragments used for measurements tended to shrink and curl, which required precise positioning on the calibration plate and a sufficiently fast measurement to avoid the effect of tissue drying, which could affect the measurement results. In the case of longer tissue fragments, it was necessary to assume a constant diameter and wall thickness for the entire analyzed sample.

Uniaxial tensile tests provide valuable information on the mechanical behavior of arterial tissue, whether for applications to intra-arterial procedures, 3D printing or deformation modeling. However, it should be noted that they do not provide a complete picture of tensile behavior and do not fully capture the complex anisotropic nature of blood vessels, which are fiber-reinforced composite materials. Arteries are composed of collagen and elastin fibers organized into helical and lamellar structures, leading to different mechanical responses depending on the direction of loading (Holzapfel et al., 2025). While uniaxial testing can approximate tensile strength in one direction, it does not account for the multidirectional load-bearing behavior observed *in vivo*. Biaxial testing can better capture physiological loading conditions and the interaction between axial and peripheral stresses (Weisbecker et al., 2012), but due to the small internal diameters of intracerebral arteries, such testing is not feasible on existing apparatus.

Furthermore, it is nearly impossible to replicate the pressures and forces that the artery experiences *in vivo*. The mechanical work of each artery is subject to variation due to the complex nature of the patient’s history, interpersonal differences, or variability in flow conditions. Furthermore, the medical field is missing clearly defined procedures or recommendations concerning the speed, timing, or forces employed during various procedures. This deficiency further complicates the formulation of clear recommendations or best practices for clinicians.

## 4 Conclusion

The study aimed to investigate the strength parameters of the major intracranial arteries (basilar artery, left and right anterior cerebral artery, left and right middle cerebral artery, anterior communicating artery) based on conducting a tensile test of anatomical specimens of human arteries. The main results of the study are the obtained values of the measured parameters: maximum Young’s modulus, E , ultimate strength, $R_{m}$ , rupture strength, $R_{u}$ , and ultimate strength, A .

Studies indicate that the least stiff arteries are the ACA, followed by the ACoA which are significantly different from the BA and MCA. They are also able to withstand higher stresses. Studies also indicate that wall thickness, which is an easily measurable parameter for clinicians with vessel wall magnetic resonance imaging, is directly related to strength parameters. The thinner the wall, the greater the elasticity and possible stresses.

Obtained data can be used in the design of new neurointerventional devices (stents, catheters, guidewires, etc.), be an essential indicator in tissue engineering for the creation of phantoms or arterial supports and provide information necessary for advanced simulation studies on cerebral blood flow and arterial wall-stent interactions.

For clinicians performing intracranial procedures, rupture strain, which illustrates how much an artery can be stretched during an intervention without risking rupture, seems most intuitive. The results suggest that stretching the artery to 7% is very unlikely to lead to rupture (which does not imply the absence of other permanent constrictions), and this limit is the same for all intracranial arteries studied and does not depend on the wall thickness and other parameters analyzed.

## Data Availability

The datasets presented in this study can be found in online repositories. The names of the repository/repositories and accession number(s) can be found in the article/Supplementary Material.
