# Supplementary material for: Statistical analysis of the measured strength parameters of the fresh main intracranial arteries
Source: Front Bioeng Biotechnol. 2025 Sep 18;13:1554674. doi: 10.3389/fbioe.2025.1554674 (PMC12488656; doi:10.3389/fbioe.2025.1554674)
Supplement: Supplementary file 1 [file Table1.docx]

Supplementary Material

1. Results of the tensile test of the cerebral arteries

| No | Artery | Sex | Age | Geometry | $\bar{d}$ (mm) | $\bar{\delta}$ (mm) | *E* (MPa) | *R_m_* (MPa) | *R_u_* (MPa) | *A* (%) |
| --- | --- | --- | --- | --- | --- | --- | --- | --- | --- | --- |
| 1 | BA | W | 20 |  | 3.48 | 0.65 | 3.56 | 0.71 | 0.58 | 25.06 |
| 2 | BA | M | 40 |  | 3.28 | 0.20 | 11.72 | 1.11 | 1.08 | 17.28 |
| 3 | BA | M | 43 |  | 4.15 | 0.62 | 1.58 | 0.39 | 0.38 | 32.34 |
| 4 | BA | M | 46 |  | 4.77 | 0.47 | 1.89 | 0.36 | 0.33 | 19.83 |
| 5 | BA | M | 51 |  | 3.13 | 0.27 | 1.29 | 0.79 | 0.77 | 16.75 |
| 6 | BA | M | 60 |  | 3.41 | 0.32 | 3.38 | 1.48 | 1.43 | 21.50 |
| 7 | BA | M | 70 |  | 2.97 | 0.42 | 5.75 | 0.94 | 0.94 | 19.06 |
| 8 | LMCA | W | 20 |  | 3.54 | 0.61 | 0.97 | 0.45 | 0.42 | 49.07 |
| 9 | LMCA | M | 40 |  | 2.09 | 0.40 | 7.73 | 1.57 | 1.31 | 23.22 |
| 10 | LMCA | M | 45 |  | 3.44 | 0.76 | 2.51 | 0.53 | 0.53 | 23.46 |
| 11 | LMCA | M | 46 |  | 2.92 | 0.20 | 4.54 | 1.03 | 0.87 | 20.28 |
| 12 | LMCA | M | 51 |  | 3.79 | 0.59 | 1.75 | 0.22 | 0.21 | 13.77 |
| 13 | LMCA | M | 54 |  | 3.65 | 0.62 | 1.83 | 0.45 | 0.45 | 26.44 |
| 14 | LMCA | M | 60 |  | 3.63 | 0.49 | 1.83 | 0.33 | 0.33 | 19.38 |
| 15 | LMCA | M | 70 |  | 2.99 | 0.28 | 7.98 | 0.95 | 0.94 | 13.68 |
| 16 | RMCA | W | 20 |  | 3.54 | 0.92 | 1.28 | 0.36 | 0.35 | 41.95 |
| 17 | RMCA | M | 40 |  | 2.59 | 0.36 | 3.43 | 0.63 | 0.59 | 17.88 |
| 18 | RMCA | M | 45 |  | 3.88 | 0.69 | 1.92 | 0.32 | 0.31 | 18.33 |
| 19 | RMCA | M | 46 |  | 1.84 | 0.27 | 1.89 | 1.03 | 1.01 | 19.61 |
| 20 | RMCA | M | 51 |  | 3.55 | 0.79 | 1.29 | 0.10 | 0.09 | 8.34 |
| 21 | RMCA | M | 54 |  | 3.52 | 0.85 | 1.35 | 0.27 | 0.26 | 22.44 |
| 22 | RMCA | M | 60 |  | 2.99 | 0.37 | 3.38 | 0.36 | 0.32 | 7.84 |
| 23 | RMCA | M | 70 |  | 3.10 | 0.33 | 7.84 | 1.07 | 1.07 | 16.62 |
| 24 | LACA | W | 20 |  | 2.29 | 0.53 | 3.44 | 0.78 | 0.76 | 30.56 |
| 25 | LACA | M | 40 |  | 1.96 | 0.20 | 11.72 | 0.88 | 0.49 | 13.56 |
| 26 | LACA | M | 46 |  | 1.94 | 0.21 | 16.14 | 3.51 | 3.48 | 24.85 |
| 27 | LACA | M | 51 |  | 3.13 | 0.35 | 7.35 | 0.80 | 0.78 | 11.12 |
| 28 | LACA | M | 60 |  | 2.59 | 0.24 | 14.09 | 2.74 | 2.70 | 22.61 |
| 29 | LACA | M | 70 |  | 1.69 | 0.14 | 13.82 | 1.35 | 1.29 | 25.72 |
| 30 | RACA | W | 20 |  | 2.30 | 0.40 | 4.05 | 1.47 | 1.45 | 51.23 |
| 31 | RACA | M | 46 |  | 2.78 | 0.35 | 18.64 | 1.51 | 1.49 | 27.56 |
| 32 | RACA | M | 51 |  | 1.79 | 0.17 | 29.06 | 2.03 | 2.01 | 7.36 |
| 33 | RACA | M | 60 |  | 2.57 | 0.34 | 5.93 | 1.17 | 1.17 | 19.61 |
| 34 | RACA | M | 70 |  | 2.50 | 0.34 | 11.49 | 1.75 | 1.70 | 19.02 |
| 35 | ACoA | M | 80 | single branching (into thicker and very thin) | 0.86 | 0.11 | 9.02 | 1.40 | 1.14 | 21.45 |
| 36 | ACoA | M | 43 | single | 1.68 | 0.12 | 7.15 | 1.44 | 1.38 | 27.51 |
| 37 | ACoA | M | 65 | single | 1.81 | 0.13 | 7.92 | 1.78 | 1.68 | 26.90 |
| 38 | ACoA | M | 43 | single | 1.83 | 0.15 | 2.71 | 1.05 | 1.02 | 40.84 |
| 39 | ACoA | W | 65 | single | 2.04 | 0.17 | 6.15 | 0.61 | 0.57 | 10.95 |
| 40 | ACoA | W | nd | single | 2.20 | 0.17 | 6.54 | 1.43 | 1.22 | 26.95 |
| 41 | ACoA | W | 55 | single | 2.20 | 0.19 | 6.83 | 1.61 | 1.57 | 24.18 |
| 42 | ACoA | M | 18 | single | 1.48 | 0.21 | 11.12 | 2.63 | 2.13 | 28.96 |
| 43 | ACoA | M | 52 | single | 1.65 | 0.21 | 11.04 | 1.61 | 1.43 | 16.72 |
| 44 | ACoA | M | 47 | double (one very thin) | 1.83 | 0.24 | 2.82 | 0.57 | 0.49 | 29.67 |
| 45 | ACoA | M | 40 | single | 1.96 | 0.26 | 7.84 | 0.45 | 0.38 | 7.39 |
| 46 | ACoA | M | 34 | double (one very thin) | 1.30 | 0.37 | 5.21 | 1.05 | 0.93 | 31.67 |
| 47 | ACoA | M | 81 | single | 1.60 | 0.42 | 3.29 | 0.57 | 0.55 | 22.15 |
| 48 | ACoA | M | 61 | double | 2.76 | 0.49 | 2.28 | 0.37 | 0.31 | 20.03 |

$\bar{d}$ – mean outer diameter, $\bar{\delta}$ – mean arterial wall thickness; $E$ – Young’s modulus; $R_{m}$ – ultimate strength; $R_{u}$ – rupture stress; $A$ – rupture strain; BA – basilar artery; LACA/RACA – left/right anterior cerebral artery; LMCA/RMCA – left/right middle cerebral artery; W – woman; M – man

1. Results of multiple comparisons
   (means, 95% percent LSD intervals, *σ_LSD_*, and homogeneous group, $HG$) for Young's modulus, *E*

| Artery | *N* (–) | $\bar{E}$ (MPa) | *σ_LSD_* (MPa) | $HG$ |
| --- | --- | --- | --- | --- |
| BA | 7 | 4.17 | 1.66 | A |
| LMCA | 8 | 3.64 | 1.56 | A |
| RMCA | 8 | 2.80 | 1.56 | A |
| LACA | 6 | 11.09 | 1.80 | B |
| RACA | 5 | 13.83 | 1.97 | B |
| ACoA | 14 | 6.42 | 1.18 | A |

1. Results of multiple comparisons
   (means, 95% percent LSD intervals, *σ_LSD_*, and homogeneous group, $HG$) for ultimate strength, $R_{m}$

| Artery | *N* (–) | $\bar{R}_{m}$ (MPa) | *σ_LSD_* (MPa) | $HG$ |
| --- | --- | --- | --- | --- |
| BA | 7 | 0.82 | 0.23 | A C |
| LMCA | 8 | 0.69 | 0.22 | A C |
| RMCA | 8 | 0.52 | 0.22 | A |
| LACA | 6 | 1.97 | 0.25 | B |
| RACA | 5 | 1.99 | 0.27 | B |
| ACoA | 14 | 1.18 | 0.16 | BC |

1. Results of multiple comparisons
   (means, 95% percent LSD intervals, *σ_LSD_*, and homogeneous group, $HG$) for rupture strength, $R_{u}$

| Artery | *N* (–) | $\bar{R}_{u}$ (MPa) | *σ_LSD_* (MPa) | $HG$ |
| --- | --- | --- | --- | --- |
| BA | 7 | 0.79 | 0.22 | A C |
| LMCA | 8 | 0.63 | 0.21 | A C |
| RMCA | 8 | 0.50 | 0.21 | A |
| LACA | 6 | 1.58 | 0.24 | B |
| RACA | 5 | 1.57 | 0.26 | B |
| ACoA | 14 | 1.06 | 0.16 | BC |
